# Knowledge, Use, and Barriers in Dyslipidemia Management: A Cross-Sectional Survey of Clinicians

**DOI:** 10.3390/jcm15072745

**Published:** 2026-04-05

**Authors:** António Mesquita-Lousada, Arsénio Barbosa, Joana Brandão Silva, Mario D’Oria, Daniela Santos Silva, José Paulo Andrade, Hugo Ribeiro, João Rocha-Neves

**Affiliations:** 1 Faculty of Medicine, University of Porto, Alameda Professor Hernâni Monteiro, 4200-319 Porto, Portugal; 2 Department of Internal Medicine, Unidade Local de Saúde do São João, EPE, 4200-319 Porto, Portugal; 3 School of Medicine and Biomedical Sciences, University of Porto, 4050-313 Porto, Portugal; 4 Marco Family Health Unit, Local Health Unit Tâmega e Sousa, 4630-219 Marco de Canaveses, Portugal; 5 Faculty of Medicine, University of Coimbra (FMUC), 3004-504 Coimbra, Portugal; 6 Division of Vascular and Endovascular Surgery, Department of Clinical Surgical and Health Sciences, University of Trieste, 34127 Trieste, Italy; 7 Palliative Care Study Center, Faculty of Medicine, University of Coimbra, 3000-548 Coimbra, Portugal; 8 RISE-Health, Department of Biomedicine, Faculty of Medicine, University of Porto, 4200-319 Porto, Portugal; 9 Department of Biomedicine, Unit of Anatomy, Faculty of Medicine, University of Porto, 4200-319 Porto, Portugal; 10 Community Palliative Care Support Team Gaia, Local Health Unit Gaia and Espinho, 4400-129 Vila Nova de Gaia, Portugal; 11 Department of Community Medicine, Health Information and Decision Making, Faculty of Medicine, University of Porto, Rua Dr. Plácido da Costa, 4200-450 Porto, Portugal; 12 Centre for Innovative Biomedicine and Biotechnology, 3004-504 Coimbra, Portugal; 13 Department of Vascular Surgery, Unidade Local de Saúde do Alto Ave, EPE, 4835-044 Guimarães, Portugal

**Keywords:** cardiovascular risk assessment, lipoprotein(a), SCORE2, LDL-C, apolipoprotein B, physician survey

## Abstract

**Introduction/Objectives:** Although contemporary guidelines strongly support intensive low-density lipoprotein cholesterol (LDL-C) lowering and the use of advanced lipid biomarkers for cardiovascular risk stratification, implementation in daily clinical practice remains inconsistent. This study aimed to assess current practices, knowledge, and perceived barriers in dyslipidemia management across medical specialties. **Methods:** We conducted a cross-sectional, anonymous online survey from August to September 2025 among physicians actively involved in lipid management. The questionnaire evaluated the use of Systematic Coronary Risk Evaluation 2 (SCORE2)-based risk assessment, familiarity with LDL-C targets, treatment intensification strategies, awareness and use of apolipoprotein B (apoB) and lipoprotein(a) [Lp(a)], perceived barriers to LDL-C goal attainment, and responses to a standardized clinical vignette. Descriptive analyses and chi-square testing were conducted. **Results:** Ninety-five physicians completed the survey, the majority practicing in Europe (92.7%), including 83.2% from Portugal (41.1% general practice/family medicine; 14.7% cardiology; 14.7% internal medicine/geriatrics; 14.7% vascular surgery; 9.5% endocrinology). SCORE2 calculators were used “often” or “always” by 52.6%, with significant inter-specialty variation (*p* < 0.001). Familiarity with LDL-C targets was high (76.8%), and 89.4% reported frequent therapy intensification when goals were not achieved; however, consistent escalation (“always”) differed markedly across specialties (*p* < 0.001). Although 69.5% were aware of recommendations for lifetime assessment of apoB/non–HDL-C/Lp(a), only 17.9% implemented them routinely. Most clinicians reported never or rarely using advanced biomarkers for residual risk assessment, and in a clinical vignette only 12.6% would consistently intensify therapy despite elevated Lp(a) and apoB (*p* = 0.004). Patient non-adherence (86.3%) was the most frequently perceived barrier. **Conclusions:** Despite the widespread awareness of LDL-C targets, important gaps persist in the consistent application of guideline-directed therapy and in the use of advanced biomarkers. The underutilization of apoB and Lp(a), together with therapeutic inertia and structural barriers, limits effective residual risk management. Bridging this gap will require coordinated efforts focused on implementation, access, and multidisciplinary care.

## 1. Introduction

Dyslipidemia, characterized by elevated levels of atherogenic lipoproteins, remains one of the most important modifiable risk factors for atherosclerotic cardiovascular disease (ASCVD) [1,2]. Cardiovascular diseases continue to represent the leading cause of mortality globally, accounting for approximately 18.6 million deaths annually, with a substantial proportion attributable to elevated low-density lipoprotein cholesterol (LDL-C) levels [2]. The causal relationship between elevated LDL-C and cardiovascular events has been firmly established through decades of epidemiological research, randomized controlled trials, and Mendelian randomization studies [3,4]. Consequently, early identification, accurate risk stratification, and optimal lipid management constitute the fundamental pillars of contemporary cardiovascular prevention strategies.

Current European guidelines on cardiovascular disease prevention, updated by the European Society of Cardiology [5] in 2021, recommend systematic cardiovascular risk assessment using the Systematic Coronary Risk Evaluation 2 (SCORE2) algorithm and afterwards its variants, SCORE2-OP for older persons and SCORE2-Diabetes for individuals with diabetes mellitus [5,6]. These updated risk prediction tools provide improved discrimination and calibration compared to the original SCORE model, incorporating geographic and demographic variations to enable personalized risk stratification [7]. The 2025 Focused Update of the European Society of Cardiology/European Atherosclerosis Society (ESC/EAS) [8] guidelines on dyslipidemia management emphasize increasingly stringent LDL-C targets stratified by cardiovascular risk category: less than 70 mg/dL (1.8 mmol/L) for high-risk patients, less than 55 mg/dL (1.4 mmol/L) for very-high-risk individuals, and less than 40 mg/dL (1.0 mmol/L) for those with recurrent cardiovascular events, with a recommended minimum 50% reduction from baseline values [9]. Additionally, these guidelines recommend lifetime assessment of advanced lipid biomarkers including apolipoprotein B (apoB), non-high-density lipoprotein cholesterol (non-HDL-C), and lipoprotein(a) [Lp(a)], recognizing their independent contribution to residual cardiovascular risk beyond conventional lipid parameters [9,10].

Despite strong evidence-based guideline recommendations supported by robust data from landmark clinical trials, real-world implementation of optimal dyslipidemia management strategies remains suboptimal across diverse healthcare settings [11,12]. Multiple large-scale registries, including the European Action on Secondary and Primary Prevention by Intervention to Reduce Events (EUROASPIRE) surveys and the DA VINCI study, have consistently documented that fewer than half of high-risk patients achieve recommended LDL-C targets, even in specialized cardiovascular care centers [11,12]. This persistent “treatment gap” between guideline recommendations and clinical practice reflects a complex interplay of multiple factors, including therapeutic inertia, uncertainty regarding treatment targets and risk stratification tools, patient non-adherence to prescribed medications, statin-associated adverse effects (both real and perceived), and insufficient awareness of the clinical utility of advanced biomarkers such as apoB and Lp(a) [13]. Previous physician surveys conducted in various international contexts have revealed considerable heterogeneity in confidence regarding cardiovascular risk estimation, knowledge of guideline-recommended LDL-C targets, and strategies for therapy intensification [14,15,16,17].

This study aimed to evaluate real-world clinical practices, barriers, and specialty-level differences in cardiovascular risk assessment, LDL-C management and the use of advanced lipid biomarkers among clinicians.

## 2. Methods

### 2.1. Study Design

This was an observational, descriptive, cross-sectional study based on an anonymous online survey distributed to physicians from multiple specialties and countries between August and September 2025.

### 2.2. Study Population and Recruitment

The survey targeted physicians, residents and specialists, in general practice/family medicine, cardiology, endocrinology, internal medicine, vascular surgery, and other specialties with active clinical practice in dyslipidemia management. Participants were recruited through institutional emails, medical professional societies, social media medical communities, and online physician networks. Inclusion criteria were: (1) a medical degree with active clinical practice in one of the specified specialties, and (2) completion of all mandatory sections of the survey. Exclusion criteria included: (1) non-physician healthcare professionals (nurses, pharmacists, and dietitians), (2) physicians without relevant clinical practice in dyslipidemia, and (3) duplicate responses (identified by metadata analysis).

### 2.3. Survey Instrument

A pilot study was initially conducted with 10 participants, including college members, cardiologists, and primary care physicians who regularly manage patients with dyslipidemia, to evaluate the feasibility and clarity of the survey. Based on their feedback, adjustments were made to the questionnaire to improve its comprehensibility and relevance.

The questionnaire was developed based on current ESC/EAS dyslipidemia guidelines [8,9], cardiovascular risk assessment tools (SCORE2 and derivatives) [5,6], and the recent literature on barriers to lipid-lowering therapy [18,19,20] (Supplementary Material-Annex S1).

Most questions used Likert-type scales, single-choice, or multiple-choice formats without logic-based branching. A brief clinical vignette was included to assess decision making in a standardized scenario. Participants were asked to respond based on the approach they considered most appropriate for the patient presented. The estimated completion time was 8 min.

### 2.4. Data Collection

The survey was administered via Google Forms (Google LLC, Mountain View, CA, USA) and disseminated via Web 2.0 platforms. Anonymity and compliance with the General Data Protection Regulation (GDPR) was ensured. An informed consent statement was presented at the beginning of the survey, explaining the study objectives, the voluntary nature of participation, and the right to withdraw at any time without penalty. No personally identifiable information was collected. Responses were stored on a secure platform with restricted access limited to the principal investigator. Upon survey closure, data were transferred to a password-protected institutional file and removed from the original platform, in accordance with academic data retention requirements.

### 2.5. Statistical Analysis

Data collection and descriptive statistics were performed using Microsoft 365 Excel version 16.107.3 (Microsoft Corp., Redmond, WA, USA). Descriptive statistics were used to analyze the survey data. Missing data was avoided, as all questions were required for submission. The pilot study results were used solely to refine the questionnaire and were excluded from the final analysis.

Data analyses were conducted using IBM SPSS Statistics (IBM Corp., release 2025. IBM SPSS Statistics for Windows, version 30.0, Armonk, NY, USA). Descriptive statistics were used to summarize participant characteristics and survey responses. Categorical variables were presented as frequencies and percentages. Responses were stratified by medical specialty to identify patterns and variations in knowledge, practices, and perceived barriers. Chi-square tests were used to assess differences across specialty groups for categorical variables, with *p* < 0.05 considered statistically significant. Given the descriptive and exploratory nature of the study, no formal hypothesis testing or multivariate analyses were performed. All analyses were conducted using SPSS version 30.0.

### 2.6. Ethical Considerations

This study adhered to the principles of the Declaration of Helsinki and complied with the GDPR. The protocol was submitted to the Ethics Committee of Faculdade de Medicina da Universidade do Porto and received approval (protocol: 356/CEFMUP-RISEHealth/2025). Participation was entirely voluntary, anonymous, and without financial compensation. Participants provided informed consent through a mandatory checkbox before beginning the survey. No risks were anticipated for the participants, and all data were handled confidentially. No identifiable data was collected, ensuring anonymity.

## 3. Results

### 3.1. Participant Characteristics

Between August and September 2025, 95 physicians completed the online survey. The majority of respondents practiced in Portugal (*n* = 79, 83.2%), with additional participants from other European countries including Italy, Romania, and Luxembourg (*n* = 9, 9.5%); Latin America comprising Brazil, Argentina, and Mexico (*n* = 4, 4.2%); North Africa/ Middle East (*n* = 1, 1.1%); South Asia (*n* = 1, 1.1%); and Oceania (*n* = 1, 1.1%).

Given the predominance of respondents from Portugal and the limited representation of other regions, geographic subgroup analyses were not performed. Subsequent inferential analyses, therefore, focused on differences across medical specialties.

Regarding medical specialty, general practice/family medicine constituted the largest group (*n* = 39, 41.1%), followed by cardiology, internal medicine, and vascular surgery (each *n* = 14, 14.7%; together, 44.2%), and endocrinology (*n* = 9, 9.5%). In terms of patient volume, 40.0% of respondents (*n* = 38) reported seeing 6–10 patients with dyslipidemia per week, 29.5% (*n* = 28) saw 11–20 patients, 14.7% (*n* = 14) saw 0–5 patients, 8.4% (*n* = 8) saw 21–30 patients, and 7.4% (*n* = 7) saw 31–40 patients weekly (Table 1).

### 3.2. Use of Cardiovascular Risk Assessment Tools

**Figure 1:**
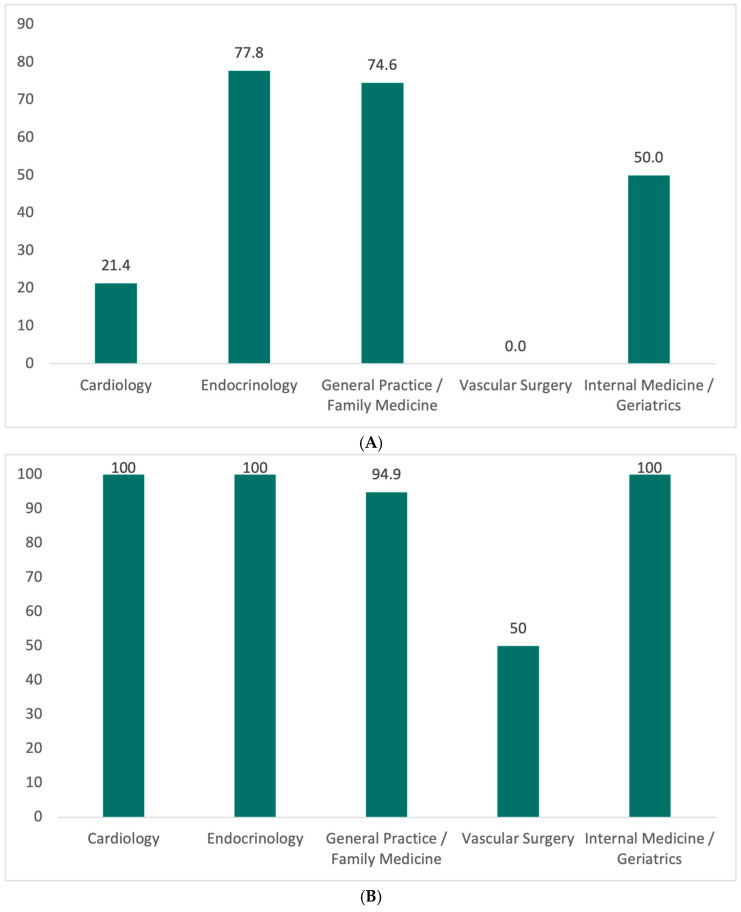
(**A**) Percentage of physicians in each specialty who reported using SCORE2 “often” or “always” for cardiovascular risk assessment. *p* < 0.001. (**B**) Percentage of physicians who reported intensifying lipid-lowering therapy “often” or “always” when LDL-C remains above guideline-recommended targets, stratified by medical specialty. *p* < 0.001. Differences between specialties were assessed using the chi-square test for categorical variables. Statistical significance was defined as *p* < 0.05.

Overall, clinicians reported frequent use of SCORE2-related calculators, with more than half (52.6%) reporting “often” or “always”, although this practice varied substantially across medical specialties (*p* < 0.001). General practice/family medicine (84.6%) and endocrinology (77.8%) demonstrated the highest adherence to systematic risk scoring, whereas vascular surgery reported the lowest (0%). A similar pattern was observed in applying SCORE2 to patients without established cardiovascular disease, with higher routine application among general practice/family medicine (79.5%) and endocrinology (66.6%), intermediate use in internal medicine/geriatrics (57.2%), and substantially lower use in cardiology (28.6%) and vascular surgery (0%) (*p* < 0.001) (Figure 1A).

Perceived barriers to achieving LDL-C targets were common and multifactorial, most frequently involving patient non-adherence (86.3%), concerns about side effects (37.9%), and delayed follow-up (21.1%), with two of these barriers differing significantly between specialties, namely patient non-adherence and delayed or absent follow-up (*p* < 0.001 and *p* = 0.02, respectively). Patient non-adherence was most frequently reported by endocrinology (100%) and internal medicine/geriatrics (100%), compared with cardiology (78.6%) and vascular surgery (78.6%). In contrast, delayed or absent follow-up was notably higher in vascular surgery (50.0%) than in general practice/family medicine (5.1%) and cardiology (42.9%).

Communication about the distinction between laboratory reference ranges and therapeutic LDL-C goals was generally well integrated into practice, where 75% of clinicians reported doing so “often” or “always”, and this behavior did not differ significantly across specialties (*p* = 0.281).

These results highlight statistically significant differences across medical specialties in the use of risk assessment tools and in selected perceived barriers to achieving LDL-C targets (Table 2).

### 3.3. Knowledge and Application of LDL-C Targets

Clinicians reported relying on multiple factors when deciding to initiate or intensify lipid-lowering therapy, with most considering formal cardiovascular risk assessment tools (78.9%), family history of premature cardiovascular disease (66.3%), and evidence of subclinical atherosclerosis (64.2%) as key determinants, whereas LDL-C levels alone guided decisions in fewer than half of the respondents (48.4%). The use of emerging biomarkers, such as IL-6 or hs-CRP, was low overall (3.2%), with no significant differences across specialties (*p* = 0.127).

There were significant differences between specialties regarding the use of SCORE2 or similar calculators (*p* < 0.001), highest in general practice/family medicine (97.4%) and internal medicine/geriatrics (85.7%), followed by cardiology (78.6%) and endocrinology (77.8%), and lowest in vascular surgery (23.4%).

Most respondents (76.8%) reported familiarity with LDL-C targets for high and very high cardiovascular risk and stated they routinely apply them. Familiarity with the targets and routine application was highest among endocrinologists (88.9%) and general practice/family medicine physicians (84.6%), followed by internists (78.6%), cardiologists (71.4%), and vascular surgeons (64.3%), with significant differences across specialties (*p* < 0.001).

Confidence in estimating the expected LDL-C reduction with statins was generally moderate to high: 67.4% of respondents reported being “confident” or “very confident,” and no significant differences were observed across specialties (*p* = 0.418).

When LDL-C targets were not achieved with statin monotherapy, most clinicians reported intensifying therapy “often” or “always” (89.4%), with significant inter-specialty variation (*p* < 0.001) (Figure 1B). Endocrinology demonstrated the highest proportion of consistent intensification (“always”: 77.8%), compared with general practice/family medicine (43.6%), internal medicine/geriatrics (35.7%), cardiology (28.6%), and vascular surgery (21.4%) (Table 3).

### 3.4. Awareness and Utilization of Advanced Lipid Biomarkers

#### 3.4.1. Awareness of Lifetime Assessment Recommendations

**Figure 2:**
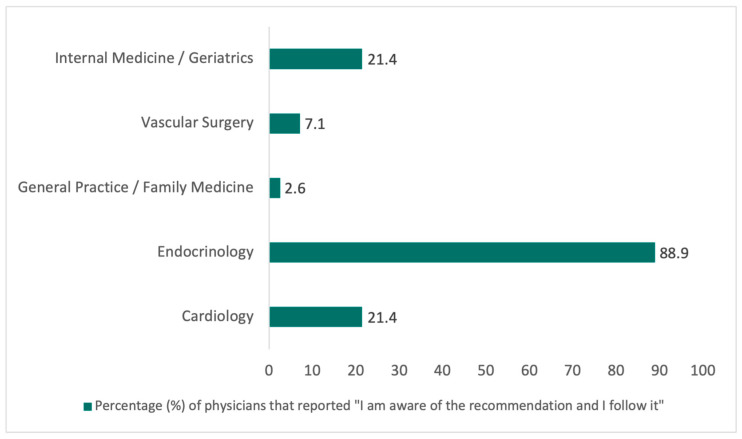
Compliance with the “once-in-a-lifetime” apoB/Lp(a) recommendation across specialties (%). Percentage of physicians who report adhering to guideline recommendations to assess apoB, non-HDL-C, or Lp(a) at least once in a lifetime across specialties. *p* < 0.001. Differences between specialties were assessed using the chi-square test. Statistical significance was defined as *p* < 0.05.

A majority of clinicians (69.5%) reported awareness of the recommendation to assess apoB, non–HDL-C, or Lp(a) at least once in a lifetime (51.6% aware but not consistently following it; 17.9% aware and following it). Adherence to this recommendation (“yes, and I follow it”) varied significantly across specialties (*p* < 0.001), being highest in endocrinology (88.9%) compared with cardiology (21.4%), internal medicine/geriatrics (21.4%), vascular surgery (7.1%), and general practice/family medicine (2.6%) (Figure 2) (Table 4).

#### 3.4.2. Use in Residual Risk Assessment

Among patients with LDL-C at target but persistent risk, 61.0% of clinicians reported never or rarely using apoB, Lp(a), or non–HDL-C to guide management, and 22.1% occasionally; only 5.3% applied these biomarkers frequently, while 21.1% selected “no access/no reimbursement” as their response. Significant inter-specialty differences were observed in response distribution (*p* = 0.004). Frequent use was reported by 14.3% of cardiologists, 11.1% of endocrinologists, 7.1% of vascular surgeons, 2.6% of general practice/family medicine physicians, and 0% of internal medicine/geriatrics physicians. Lack of access or reimbursement was most frequently reported in general practice/family medicine (38.5%) and endocrinology (22.2%).

In primary prevention risk stratification, among clinicians using imaging-based tools (e.g., carotid or femoral ultrasound and coronary calcium scoring), 11.6% reported never using them, 32.6% reported rare use, 38.9% reported occasional use, and 16.8% reported routine use. No significant differences were observed across medical specialties in the distribution of responses (*p* = 0.812) (Table 4).

#### 3.4.3. Clinical Vignette: Elevated Lp(a) and ApoB Despite LDL-C at Target

Participants were presented with a clinical scenario of a 60-year-old patient on high-intensity statin with LDL-C at target (58 mg/dL) but elevated Lp(a) (65 mg/dL) and apoB (85 mg/dL). In this hypothetical scenario, 35.8% of clinicians reported not assessing Lp(a)/apoB in routine practice; 20.0% would not intensify therapy unless LDL-C was also above target; 31.6% would intensify therapy if additional risk factors were present; and 12.6% would always intensify lipid-lowering therapy. Responses differed significantly across specialties (*p* = 0.004). The proportion selecting “yes, always” was highest among internal medicine/geriatrics (28.6%) and cardiology (21.4%), compared with endocrinology (11.1%), general practice/family medicine (7.7%), and vascular surgery (0%). The highest proportion selecting “not applicable/I do not assess Lp(a)/apoB” was observed in general practice/family medicine (59.0%) (Table 4).

#### 3.4.4. Perceived Utility of Biomarkers

When asked which additional biomarkers helped manage cardiovascular risk, triglycerides/remnant cholesterol were most frequently selected (58.9%), followed by Lp(a) (47.4%) and apoB (36.8%). Significant inter-specialty differences were observed for apoB (*p* < 0.001) and Lp(a) (*p* = 0.005). ApoB was considered helpful by 100.0% of endocrinologists, compared with 50.0% of internal medicine/geriatrics physicians, 30.8% of general practice/family medicine physicians, 28.6% of vascular surgeons, and 14.3% of cardiologists. Lp(a) was considered helpful by 100.0% of endocrinologists, compared with 57.1% of cardiologists, 50.0% of vascular surgeons and internal medicine/geriatrics physicians, and 28.2% of general practice/family medicine physicians. Use of hs-CRP (23.2%, *p* = 0.262), triglycerides/remnant cholesterol (58.9%, *p* = 0.252), IL-6 (7.4%, *p* = 0.869), and selection of “none of the above” (13.7%, *p* = 0.163) did not differ significantly across specialties (Table 4).

## 4. Discussion

### 4.1. Main Findings

In this multicenter cross-sectional survey of 95 clinicians actively involved in dyslipidemia management, frequent use of SCORE2-related cardiovascular risk calculators and generally good familiarity with guideline-recommended LDL-C targets were observed, yet there was a marked heterogeneity across specialties in how these tools are applied and translated into treatment decisions. While most respondents reported routinely applying LDL-C goals for high- and very-high-risk patients and nearly 90% stated they often or always intensified lipid-lowering therapy when targets were not achieved, the integration of advanced lipid biomarkers into clinical practice remained limited: although approximately two thirds were aware of recommendations for lifetime assessment of apoB, non-HDL-C, or Lp(a), fewer than one in five consistently followed them, and 61% reported never or rarely using these biomarkers to guide residual risk management, with one fifth citing lack of access or reimbursement. In the clinical vignette of a patient with LDL-C at target but elevated apoB and Lp(a), more than one-third of clinicians indicated they do not assess these biomarkers in routine practice, and one in five would not intensify therapy unless LDL-C was also above target, underscoring an important gap between contemporary ESC/EAS guidance on residual risk and real-world therapeutic strategies. Across specialties, patient non-adherence, concerns about statin side effects, and delayed or absent follow-up emerged as the most frequently perceived barriers to achieving LDL-C goals, highlighting that both knowledge–practice discrepancies and structural constraints contribute to the suboptimal implementation of evidence-based dyslipidemia care.

### 4.2. Risk Assessment Tools: The SCORE2 Implementation Gap

Despite the 2021 ESC guidelines and the recent 2025 update recommending SCORE2 and its variants for systematic cardiovascular risk evaluation [5,8], present findings indicate that adoption remains incomplete, and are compatible with evidence reporting the variable uptake of risk calculators in clinical practice [13,16,21].

Several factors may explain the limited adoption of SCORE2. First, time constraints in busy clinical settings may discourage calculator use, particularly in primary care where consultation times are limited [22]. Second, physicians may rely on clinical gestalt rather than formal risk estimation, especially when managing patients with established cardiovascular disease or obvious high-risk features [23]. Third, the transition from SCORE to SCORE2 requires familiarity with updated risk categories and geographic calibration, which may create temporary implementation barriers [7]. Finally, electronic health record integration of these calculators remains heterogeneous across healthcare systems, with many requiring manual data entry [24]. Several studies examining the implementation of cardiovascular risk calculators have identified multiple barriers to their routine use, including limited familiarity with the tools, time constraints during consultations, the lack of integration into electronic health record systems, and variable clinician acceptance of algorithm-based risk estimation [25,26].

### 4.3. Knowledge and Application of LDL-C Targets

Overall, clinicians reported a risk-based rather than LDL-C-centric approach to treatment decisions, with most considering formal risk scores, family history, and subclinical atherosclerosis, and fewer than half relying primarily on LDL-C levels alone. This pattern is broadly consistent with ESC/EAS and AHA/ACC recommendations that anchor treatment intensity in global cardiovascular risk. Still, it may also signal the under-recognition of LDL-C’s central causal role, particularly in very-high-risk patients for whom aggressive lowering is recommended regardless of other factors [8,17,19]. High confidence in estimating expected LDL-C reductions with statins across specialties further suggests that, at a conceptual level, key elements of guideline-based LDL-C management are well disseminated. Given the consistently low LDL-C target attainment reported in large registries such as EUROASPIRE and DA VINCI [11,12], these self-reported intensification rates likely overestimate actual practice and highlight the well-described gap between perceived and actual guideline implementation. Taken together, the present findings suggest that, while numerical LDL-C targets and statin potency are widely known and clinicians express high confidence in their use, therapeutic inertia and the underuse of combination lipid-lowering therapy remain key barriers to achieving guideline-recommended LDL-C control in real-world settings.

### 4.4. Barriers to Target Achievement: A Multifaceted Challenge

Patient non-adherence was the dominant barrier (86.3%), consistent with the extensive literature documenting medication adherence challenges in chronic disease management [27]. Statin adherence rates typically decline to 40–60% within 1 year of initiation, driven by factors such as the lack of symptoms in primary prevention, concerns about side effects, and the complexity of medication regimens [28]. The finding that 37.9% of clinicians reported concerns about side effects as a barrier underscores the role of the nocebo effect and emphasizes the importance of proactive, empathetic communication about statin safety. Large individual participant data meta-analyses of double-blind randomized trials have demonstrated that, aside from muscle-related events and a modest increase in incident diabetes, statins are not causally associated with most of the adverse effects listed on product labels, including cognitive, psychiatric, or renal events [29]. This suggests that the perceived burden of statin-related side effects may exceed the true pharmacological risk. Delayed or absent follow-up (21.1%) and statin intolerance (21.1%) represent additional actionable barriers. Several investigations have identified multiple drivers of non-adherence, including patients’ preference for lifestyle modifications over pharmacological treatment, general aversion to long-term medication, concerns about polypharmacy, and fear of potential adverse effects. In addition, structural and healthcare system factors, such as limited patient education, insufficient clinician–patient communication, and the complexity of treatment regimens, may further contribute to the discontinuation or irregular use of lipid-lowering therapy [30,31,32].

The relatively lower reported use of SCORE2 among cardiologists in this cohort may reflect the fact that cardiology practice frequently involves patients with established atherosclerotic cardiovascular disease or those already classified as very high cardiovascular risk, in whom formal primary prevention risk estimation tools are less relevant for guiding treatment decisions [33,34]. Systematic follow-up protocols, including reminder systems and patient navigation programs, can improve retention in care [35]. For patients with genuine statin intolerance, strategies include dose reduction, alternate-day dosing, switching to hydrophilic statins, or non-statin alternatives (ezetimibe, bempedoic acid, and PCSK9 inhibitors) [8,36].

Cost or access to medication (18.9%) and uncertainty about guideline targets (7.4%) were less frequently cited but important. Healthcare system-level interventions, including generic statin availability, medication assistance programs, and formulary optimization, can address financial barriers [23]. Continuing medical education that emphasizes guideline updates and practical implementation strategies can reduce uncertainty [37].

### 4.5. Advanced Lipid Biomarkers: The Missed Opportunity of apoB and Lp(a)

Barriers to biomarker utilization include limited awareness and the absence of specific Lp(a)-lowering therapies, although antisense oligonucleotides and small interfering RNAs are in advanced development [38]. Laboratory availability and reimbursement issues [39] are other known obstacles to biomarker use. Advocacy for universal Lp(a) screening, as recently endorsed by the Canadian Cardiovascular Society and other organizations [39], coupled with education on its clinical implications, may improve uptake.

The findings are particularly concerning given the robust evidence base supporting these markers. Apolipoprotein B provides a direct measure of atherogenic particle number and offers superior cardiovascular risk prediction compared with LDL-C alone, particularly in patients with hypertriglyceridemia, diabetes, or metabolic syndrome [40,41]. ApoB accounts for discordance between LDL-C and particle number, identifying individuals at higher-than-expected risk based on LDL-C alone [42].

Lipoprotein(a) is an independent, genetically determined, causal risk factor for atherosclerotic cardiovascular disease and aortic stenosis, affecting approximately 20% of the global population at elevated levels (>50 mg/dL) [43]. Mendelian randomization studies confirm its causal role, independent of LDL-C [44]. Current guidelines recommend measuring Lp(a) at least once to identify individuals who may benefit from more intensive LDL-C lowering and closer monitoring [9]. The clinical vignette in the survey presented a patient with LDL-C at target but elevated Lp(a) and apoB, revealing that 35.8% did not assess these biomarkers at all, and only 12.6% would always intensify therapy in this scenario. This represents a significant missed opportunity to modify residual risk.

### 4.6. Specialty Differences: Implications for Collaborative Care

Significant heterogeneity across medical specialties was observed in multiple domains. Endocrinologists demonstrated the highest adherence to guideline recommendations, including SCORE2 use (77.8% often), advanced biomarker assessment (88.9% aware and following apoB/Lp(a) recommendations), and therapy intensification (77.8% always). Conversely, vascular surgeons showed lower rates of systematic risk assessment (50% never use SCORE2) and intensification (21.4% always).

These differences likely reflect specialty-specific training, patient populations, and clinical focus. Endocrinologists routinely manage complex metabolic conditions that require meticulous lipid control, thereby fostering familiarity with advanced strategies [45]. The integration of specialty expertise through shared care pathways, teleconsultation for complex cases, and interdisciplinary education can harmonize practices and improve outcomes [46]. Patients with vascular surgery or cardiology conditions may particularly benefit from referral to lipid clinics or endocrinology for the optimization of medical therapy.

### 4.7. Strengths and Limitations

This study has several strengths. It is among the first international surveys to comprehensively assess physician knowledge, practices, and barriers across multiple dimensions of dyslipidemia management, including risk assessment tools (SCORE2), advanced biomarkers (apoB and Lp(a)). The inclusion of multiple specialties (general practice, cardiology, endocrinology, and vascular surgery) provides a multifaceted perspective on current practice patterns. The use of a clinical vignette alongside knowledge questions offers insights into real-world decision making. A limitation of this study is the limited characterization of respondents. Although medical specialty was recorded, the survey did not collect additional information such as years of clinical experience, training background or certifications, or characteristics of the practice setting (e.g., type or size of institution). These variables could have provided additional context and enabled more detailed stratified analyses of clinical practice patterns. Finally, the survey did not include specific questions exploring the reasons underlying the limited use of SCORE2 risk calculators among respondents, which prevents a deeper understanding of the barriers influencing this practice.

However, important limitations must be acknowledged. First, the sample size (*N* = 95) and geographic concentration (83.2% from Iberia) limit generalizability to broader international contexts. Although most respondents were currently practicing in Portugal, it is important to note that the majority of Portuguese specialists in vascular surgery and anesthesiology are required to complete formal international training periods as part of their residency curriculum. As such, many participants have worked or trained abroad, thereby enhancing the external validity and international relevance of their perspectives despite their current geographic location. Cultural, healthcare system, and reimbursement differences across countries may influence practices in ways not captured here [11,47,48]. Second, the survey was disseminated through the authors’ professional networks and social media platforms, which may have excluded professionals not connected to these networks or those less active online, leading to network bias. Additionally, some healthcare professionals who do not engage with Web 2.0 platforms might have been excluded from the survey, further limiting the generalizability of the findings [49]. Third, selection bias is inherent in voluntary online surveys; respondents may be more engaged, knowledgeable, or motivated than non-respondents, potentially overestimating overall awareness and adherence [50]. Fourth, self-report is subject to social desirability bias, with physicians potentially overreporting guideline-concordant behaviors [51].

Validation against objective measures (e.g., prescribing data and patient lipid control rates) would strengthen the conclusions but was beyond the scope of this study. Finally, the survey did not assess patient-level factors (e.g., preferences, health literacy, and socioeconomic status) that critically influence outcomes in dyslipidemia management [52].

In contrast to the previous survey by Barter et al., which highlighted heterogeneity in dyslipidemia management practices, this study includes a truly multidisciplinary cohort of vascular surgeons, anesthesiologists, cardiologists, intensivists, and internists. It applies a vascular surgery-specific clinical scenario, providing insights that have not been previously explored [16]. Additionally, the granular analysis of perioperative pharmacological strategies and the European clinical context coupled with statistical associations between specialty, experience, and decision making offer new perspectives that extend beyond the heterogeneity already described in earlier work.

## 5. Conclusions

In this multi-specialty survey of clinicians involved in dyslipidemia management, a persistent implementation gap between evidence-based recommendations and real-world practice was identified. While awareness of LDL-C targets and reported use of risk-based approaches were generally high, consistent therapeutic intensification and systematic application of advanced lipid biomarkers remain suboptimal. In particular, the limited use of apoB and Lp(a), despite strong guideline endorsement and robust causal evidence, highlights an underexploited opportunity to identify residual risk and more precisely target cardiovascular prevention.

Marked heterogeneity across specialties further underscores that the dissemination of guidelines alone is insufficient to ensure uniform adoption. Structural barriers, including access and reimbursement constraints, coexist with therapeutic inertia and patient-level challenges, collectively limiting optimal lipid control.

Bridging this gap will require coordinated strategies integrating targeted education, electronic decision-support tools, multidisciplinary collaboration, and policy-level interventions to expand access to advanced diagnostics and therapies. As cardiovascular prevention enters an era of increasingly personalized risk stratification, the systematic implementation of validated tools, ranging from SCORE2 to apoB and Lp(a), will be essential to translating scientific advances into measurable reductions in the burden of atherosclerotic cardiovascular disease.

## Figures and Tables

**Table 1:** Participant demographics and clinical practice characteristics.

	Total *N* = 95 (%)
**Country/Region**	
Portugal	79 (83.2)
Other European countries	9 (9.5)
Latin America	4 (4.2)
North Africa/Middle East	1 (1.1)
South Asia	1 (1.1)
Oceania	1 (1.1)
**What is your practicing specialty?**	
General Practice/Family	39 (41.1)
Cardiology	14 (14.7)
Internal Medicine/Geriatrics	14 (14.7)
Vascular Surgery	14 (14.7)
Endocrinology	9 (9.5)
Others *	5 (1.1)
**Number of patients with dyslipidemia seen per week**	
0–5	14 (14.7)
6–10	38 (40.0)
11–20	28 (29.5)
21–30	8 (8.4)
31–40	7 (7.4)

Other European countries—Italy, Romania, Luxembourg; Latin America—Brazil, Argentina, Mexico; North Africa/Middle East—Egypt; South Asia—India; Oceania—Australia. Statistical comparisons between specialties were performed using the chi-square test. A *p* < 0.05 was considered statistically significant. * Others include: Nephrology, Oncology, Rheumatology, General Surgery, and Occupational Medicine.

**Table 2:** Risk assessment practices and LDL-C management by medical specialty.

	Total *N* (%) *N* = 95	Cardiology *n* = 14	Endocrinology *n* = 9	General Practice/Family Medicine *n* = 39	Vascular Surgery *n* = 14	Internal Medicine/ Geriatrics *n* = 14	*p* Value
**How often do you use SCORE2, SCORE2-OP, or SCORE2-Diabetes calculators for cardiovascular risk assessment?**							
Never	14 (14.7)	3 (21.4)	0 (0.0)	1 (2.6)	7 (50.0)	1 (7.1)	<0.001
Rarely	11 (11.6)	5 (35.7)	0 (0.0)	1 (2.6)	4 (28.6)	1 (7.1)	
Sometimes	20 (21.1)	3 (21.4)	2 (22.2)	4 (10.3)	3 (21.4)	5 (35.7)	
Often	24 (25.3)	2 (14.3)	7 (77.8)	10 (25.6)	0 (0.0)	5 (35.7)	
Always	26 (27.4)	1 (7.1)	0 (0.0)	23 (59.0)	0 (0.0)	4 (14.3)	
**How often do you apply the SCORE2 tool in patients without known cardiovascular disease?**							
Never	12 (12.6)	1 (7.1)	0 (0.0)	1 (2.6)	7 (50.0)	1 (7.1)	<0.001
Rarely	8 (8.4)	3 (21.4)	0 (0.0)	0 (0.0)	3 (21.4)	1 (7.1)	
Only in selected cases	26 (27.4)	6 (42.9)	3 (33.3)	7 (17.9)	4 (28.6)	4 (28.6)	
For every patient at least once	15 (15.8)	4 (28.6)	3 (33.3)	4 (10.3)	0 (0.0)	4 (28.6)	
Routinely	34 (35.8)	0 (0.0)	3 (33.3)	27 (69.2)	0 (0.0)	4 (28.6)	
**What are the most common barriers to achieving LDL-C targets in your practice?**							
Patient non-adherence	82 (86.3)	11 (78.6)	9 (100)	37 (94.9)	11 (78.6)	14 (100)	<0.001
Concerns about side effects	36 (37.9)	1 (7.1)	5 (55.6)	19 (48.7)	5 (35.7)	3 (21.4)	0.043
Delayed or absent follow-up	20 (21.1)	6 (42.9)	3 (33.3)	2 (5.1)	7 (50.0)	2 (14.3)	0.02
Statin intolerance	20 (21.1)	1 (7.1)	3 (33.3)	19 (28.7)	5 (35.7)	3 (21.4)	0.306
Cost or access to medication	18 (18.9)	1 (7.1)	2 (22.2)	11 (28.2)	1 (7.1)	3 (21.4)	0.023
Lack of clear communication with patients	13 (13.7)	5 (35.7)	0 (0.0)	2 (5.1)	2 (14.3)	4 (28.6)	0.676
Uncertainty about guideline targets	7 (7.4)	1 (7.1)	0 (0.0)	2 (5.1)	1 (7.1)	2 (14.3)	0.837
Insufficient statin potency	21 (22.1)	2 (14.3)	3 (33.3)	6 (15.4)	5 (35.7)	5 (35.7)	0.251
**Do you explain to patients that laboratory reference values may differ from therapeutic targets?**							
Never	3 (3.2)	0 (0.0)	0 (0.0)	0 (0.0)	1 (7.1)	1 (7.1)	0.281
Rarely	7 (7.4)	2 (14.3)	0 (0.0)	3 (7.7)	1 (7.1)	1 (7.1)	
Sometimes	14 (14.7)	3 (21.4)	1 (11.1)	3 (7.7)	2 (14.3)	4 (28.6)	
Often	28 (29.5)	2 (14.3)	1 (11.1)	13 (33.3)	7 (50.0)	4 (28.6)	
Always	43 (45.3)	7 (50.0)	7 (77.8)	20 (51.3)	3 (21.4)	4 (28.6)	

Abbreviations: SCORE2—Systematic Coronary Risk Evaluation 2; SCORE2-OP—Systematic Coronary Risk Evaluation 2-Older Persons; SCORE2-Diabetes—Systematic Coronary Risk Evaluation 2-Diabetes; LDL-C—low-density lipoprotein cholesterol. Differences between specialties were assessed using the chi-square test for categorical variables. Statistical significance was defined as *p* < 0.05.

**Table 3:** Factors guiding lipid-lowering therapy decisions and confidence in LDL-C reduction estimates.

	Total N (%) *N* = 95	Cardiology *n* = 14	Endocrinology *n* = 9	General Practice/Family Medicine *n* = 39	Vascular Surgery *n* = 14	Internal Medicine/Geriatrics *n* = 14	*p* Value
**What factors do you consider when deciding to initiate or intensify lipid-lowering therapy?**							
LDL-C level alone	46 (48.4)	7 (50.0)	5 (55.6)	15 (38.5)	9 (64.3)	8 (57.1)	0.59
SCORE2 or other risk calculator	75 (78.9)	11 (78.6)	7 (77.8)	38 (97.4)	3 (23.4)	12 (85.7)	<0.001
Family history of early cardiovascular disease	63 (66.3)	11 (78.6)	7 (77.8)	25 (64.1)	5 (64.3)	9 (64.3)	0.676
Subclinical atherosclerosis (e.g., coronary calcium…)	61 (64.2)	11 (78.6)	6 (66.7)	25 (64.1)	9 (64.3)	8 (57.1)	0.722
Lp(a) and apoB	0 (0.0)	0 (0.0)	0 (0.0)	0 (0.0)	0 (0.0)	0 (0.0)	-
Patient preferences	29 (30.5)	6 (42.9)	1 (11.1)	15 (38.5)	1 (7.1)	6 (42.9)	0.061
Emerging biomarkers (e.g., Il-6, hs-CRP)	3 (3.2)	0 (0.0)	0 (0.0)	0 (0.0)	1 (7.1)	2 (14.3)	0.127
**Are you familiar with the recommended LDL-C targets for high and very high risk?**							
Somewhat familiar	2 (2.1)	0 (0.0)	0 (0.0)	0 (0.0)	0 (0.0)	0 (0.0)	<0.001
Yes, but I do not consistently apply them	20 (21.1)	4 (28.6)	1 (11.1)	6 (15.4)	5 (35.7)	3 (21.4)	
Yes, and I apply them routinely	73 (76.8)	10 (71.4)	8 (88.9)	33 (84.6)	9 (64.3)	11 (78.6)	
**How confident are you in estimating the expected LDL-C reduction with statin therapy?**							
Not at all confident	2 (2.1)	0 (0.0)	0 (0.0)	0 (0.0)	1 (7.1)	0 (0.0)	0.418
Not confident	5 (5.3)	2 (14.3)	0 (0.0)	1 (2.6)	1 (7.1)	1 (7.1)	
Somewhat confident	24 (25.3)	2 (14.3)	2 (22.2)	9 (23.1)	4 (28.6)	6 (42.9)	
Confident	43 (45.3)	6 (42.9)	4 (44.4)	20 (51.3)	7 (50.0)	4 (28.6)	
Very confident	21 (22.1)	4 (28.6)	3 (33.3)	9 (23.1)	1 (7.1)	3 (21.4)	
**When LDL-C targets are not reached with statin monotherapy, how often do you intensify therapy (e.g., increase dose, add ezetimibe)?**							
Never	1 (1.1)	0 (0.0)	0 (0.0)	0 (0.0)	0 (0.0)	0 (0.0)	<0.001
Rarely	2 (2.1)	0 (0.0)	0 (0.0)	0 (0.0)	2 (14.3)	0 (0.0)	
Sometimes	7 (7.4)	0 (0.0)	0 (0.0)	2 (5.1)	5 (35.7)	0 (0.0)	
Often	48 (50.5)	10 (71.4)	2 (11.1)	20 (51.3)	4 (28.6)	9 (64.3)	
Always	37 (38.9)	4 (28.6)	7 (77.8)	17 (43.6)	3 (21.4)	5 (35.7)	

Abbreviations: LDL-C—low-density lipoprotein cholesterol; SCORE2—Systematic Coronary Risk Evaluation 2; Lp(a)—lipoprotein(a); apoB—apolipoprotein B; hs-CRP—high-sensitivity C-reactive protein; IL-6—interleukin 6. Differences between specialties were assessed using the chi-square test. Statistical significance was defined as *p* < 0.05.

**Table 4:** Use of advanced lipid biomarkers and risk reclassification tools.

	Total N (%) *N* = 95	Cardiology *n* = 14	Endocrinology *n* = 9	General Practice/Family Medicine *n* = 39	Vascular Surgery *n* = 14	Internal Medicine/ Geriatrics *n* = 14	*p* Value
**Are you aware of the recommendation to assess apoB, non-HDL-C, or Lp(a) at least once in a lifetime?**							
No	29 (30.5)	1 (7.1)	0 (0.0)	16 (41.0)	7 (50.0)	2 (14.3)	<0.001
Yes, but I do not follow it	49 (51.6)	10 (71.4)	1 (11.1)	22 (56.4)	6 (42.9)	9 (64.3)	
Yes, and I follow it	17 (17.9)	3 (21.4)	8 (88.9)	1 (2.6)	1 (7.1)	3 (21.4)	
**In patients with LDL-C at target but persistent risk, how often do you use ApoB/Lp(a) or non-HDL-C to guide management?**							
Never	26 (27.4)	3 (21.4)	0 (0.0)	9 (23.1)	8 (57.1)	3 (21.4)	0.004
Rarely	23 (24.2)	6 (42.9)	3 (33.3)	7 (17.9)	3 (21.4)	2 (14.3)	
Occasionally	21 (22.1)	1 (7.1)	3 (33.3)	7 (17.9)	2 (14.3)	8 (57.1)	
Yes, frequently	5 (5.3)	2 (14.3)	1 (11.1)	1 (2.6)	1 (7.1)	0 (0.0)	
No access/No reimbursement	20 (21.1)	2 (14.3)	2 (22.2)	15 (38.5)	0 (0.0)	1 (7.1)	
**Do you request carotid ultrasound, femoral ultrasound, or coronary calcium score to help stratify risk in primary prevention?**							
Never	11 (11.6)	1 (7.1)	2 (22.2)	5 (12.8)	1 (7.1)	1 (7.1)	0.812
Rarely	31 (32.6)	4 (28.6)	3 (33.3)	15 (38.5)	3 (21.4)	4 (28.6)	
Occasionally	37 (38.9)	5 (35.7)	4 (44.4)	15 (38.5)	6 (42.9)	5 (35.7)	
Routinely	16 (16.8)	4 (28.6)	0 (0.0)	4 (10.3)	4 (28.6)	4 (28.6)	
**In your clinical practice, would you intensify lipid-lowering therapy in this** scenario? *							
Not applicable/I do not assess Lp(a)/apoB	34 (35.8)	2 (14.3)	1 (11.1)	23 (59.0)	2 (14.3)	4 (28.6)	0.004
No, only if LDL-C is also above target	19 (20.0)	3 (21.4)	2 (22.2)	4 (10.3)	8 (57.1)	1 (7.1)	
Yes, if other risk factors are present	30 (31.6)	6 (42.9)	5 (55.6)	9 (23.1)	4 (28.6)	5 (35.7)	
Yes, always	12 (12.6)	3 (21.4)	1 (11.1)	3 (7.7)	0 (0.0	4 (28.6)	
**Which additional biomarkers do you consider useful in managing cardiovascular risk?**							
ApoB	35 (36.8)	2 (14.3)	9 (100.0)	12 (30.8)	4 (28.6)	7 (50.0)	<0.001
Lp(a)	45 (47.4)	8 (57.1)	9 (100.0)	11 (28.2)	7 (50.0)	7 (50.0)	0.005
hs-CRP	22 (23.2)	5 (35.7)	4 (44.4)	7 (17.9)	4 (28.6)	2 (14.3)	0.262
Triglycerides/remnant cholesterol	56 (58.9)	6 (42.9)	8 (88.9)	24 (61.5)	6 (42.9)	9 (64.3)	0.252
IL-6	7 (7.4)	1 (7.1)	1 (11.1)	2 (5.1)	2 (14.3)	1 (7.1)	0.869
None of the above	13 (13.7)	4 (28.6)	0 (0.0)	6 (15.4)	3 (21.4)	0 (0.0)	0.163

* Clinical scenario: A 60-year-old patient without established ASCVD on high-intensity statin therapy (e.g., rosuvastatin 20 mg) has LDL-C 58 mg/dL (i.e., at guideline target for their risk category). Laboratory results show Lp(a) = 65 mg/dL (≥50 mg/dL) and apoB = 85 mg/dL (≥65–80 mg/dL); non-HDL-C 98 mg/dL; triglycerides 150 mg/dL. Renal and hepatic function are normal; adherence is confirmed. Abbreviations: LDL-C—low-density lipoprotein cholesterol; Lp(a)—lipoprotein(a); apoB—apolipoprotein B; non-HDL-C—non–high-density lipoprotein cholesterol. Differences between specialties were assessed using the chi-square test. Statistical significance was defined as *p* < 0.05.

## Data Availability

The data supporting the findings of this study are available from the corresponding author upon reasonable request.

## Supplementary Materials

The following supporting information can be downloaded at https://www.mdpi.com/article/10.3390/jcm15072745/s1: Annex S1: Survey instrument used to assess physicians’ knowledge, practices, and barriers in dyslipidemia management.
