# Supplementary material for: Knowledge, Use, and Barriers in Dyslipidemia Management: A Cross-Sectional Survey of Clinicians
Source: J Clin Med. 2026 Apr 5;15(7):2745. doi: 10.3390/jcm15072745 (PMC13072749; doi:10.3390/jcm15072745)
Supplement: Supplementary file 1 [file jcm-15-02745-s001.zip › jcm-4187212-supplementary.pdf]

Supplemental Material – Annex S1

Knowledge, Use, and Barriers in Dyslipidemia Management

Dear colleague,

You are invited to participate in a research study aimed at exploring medical doctors' knowledge, clinical experience, and perceptions regarding the assessment and treatment of dyslipidemia in clinical practice.

This questionnaire, containing 16 questions, is part of a European scientific project that seeks to analyze current practices in lipid profile testing, the therapeutic strategies adopted in response to elevated levels, and the main barriers to its routine implementation in the context of atherosclerotic cardiovascular disease prevention and management.

Participation and Data Handling

Participation in this survey is voluntary and takes approximately 10 minutes. The questionnaire includes multiple-choice and open-ended questions related to your clinical practice and decision making concerning the use of the lipid profile as a cardiovascular risk marker.

All data will be collected anonymously and used exclusively for scientific purposes. They will be analyzed in aggregate form, with no possibility of individual identification. The study fully complies with the General Data Protection Regulation (GDPR), ensuring confidentiality, security, and data integrity.

All the data will be deleted in August 2026. The investigators declare that they have no conflicts of interest related to this research project.

By proceeding with the questionnaire, you are providing your **informed consent** to participate in this study. You may withdraw at any time or request the deletion of your data by contacting the research team at: **jneves@med.up.pt**.

Demographics

Country:

- Portugal
- Spain
- Italy
- Brazil
- Other, please specify

What is your medical speciality?

- Cardiology
- Endocrinology
- Internal Medicine
- General Practice/Family Medicine
- Nephrology
- Other, please specify

How many patients do you encounter with dyslipidemia per week in your practice?

- 0 to 5
- 6 to 10
- 11 to 20
- 21 to 30
- 31 to 40

Section 1

1. How often do you use SCORE2, SCORE2-OP, or SCORE2-Diabetes calculators for cardiovascular risk assessment?

- o Never
- o Rarely
- o Sometimes
- o Often
- o Always

2. How often do you apply the SCORE2 tool in patients without known cardiovascular disease?

- o Never
- o Rarely
- o Only in selected cases
- o For every patient at least once
- o Routinely

3. What are the most common barriers to achieving LDL-C targets in your practice? (You can select multiple answers)

- o Patient non-adherence
- o Concerns about side effects
- o Delayed or absent follow-up
- o Statin intolerance
- o Cost or access to medication
- o Lack of clear communication with patients
- o Uncertainty about guideline targets
- o Insufficient statin potency

4. Do you explain to patients that laboratory reference values may differ from therapeutic targets?

- o Never
- o Rarely
- o Sometimes
- o Often
- o Always

Section 2

5. What factors do you consider when deciding to initiate or intensify lipid-lowering therapy? (You can select multiple answers)

- LDL-C level alone
- SCORE2 or other risk calculator
- Family history of early cardiovascular disease
- Subclinical atherosclerosis (e.g., coronary calcium…)
- Lp(a) and apoB
- Patient preferences
- Emerging biomarkers (e.g., Il-6, hs-CRP)

6. Are you familiar with the recommended LDL-C targets for high and very high risk?

- Somewhat familiar
- Yes, but I do not consistently apply them
- Yes, and I apply them routinely

7. How confident are you in estimating the expected LDL-C reduction with statin therapy?

- Not at all confident
- Not confident
- Somewhat confident
- Confident
- Very confident

8. When LDL-C targets are not reached with statin monotherapy, how often do you intensify therapy (e.g., increase dose, add ezetimibe)?

- Never
- Rarely
- Sometimes
- Often
- Always

Section 3

9. Are you aware of the recommendation to assess apoB, non-HDL-C, or Lp(a) at least once in a lifetime?

- o No
- o Yes, but I do not follow it
- o Yes, and I follow it

10. In patients with LDL-C at target but persistent risk, how often do you use ApoB/Lp(a) or non-HDL-C to guide management?

- o Never
- o Rarely
- o Occasionally
- o Yes, frequently
- o No access / No reimbursement

11. Do you request carotid ultrasound, femoral ultrasound, or coronary calcium score to help stratify risk in primary prevention?

- o Never
- o Rarely
- o Occasionally
- o Routinely

12. A 60-year-old patient without established ASCVD, on high-intensity statin therapy (e.g., rosuvastatin 20 mg), has LDL-C 58 mg/dL (i.e., at guideline target for their risk category). Laboratory results show Lp(a) = 65 mg/dL (≥50 mg/dL) and apoB = 85 mg/dL (≥65–80 mg/dL); non-HDL-C 98 mg/dL; triglycerides 150 mg/dL. Renal and hepatic function are normal; adherence is confirmed.

In your clinical practice, would you intensify lipid-lowering therapy in this scenario?

- o Not applicable / I do not assess Lp(a)/apoB
- o No, only if LDL-C is also above target
- o Yes, if other risk factors are present
- o Yes, always

13. Which additional biomarkers do you consider useful in managing cardiovascular risk? (You can select multiple answers)

- o ApoB
- o Lp(a)

- hs-CRP
- Triglycerides / remnant cholesterol
- IL-6
- None of the above
